# Biodistribution of extracellular vesicles following administration into animals: A systematic review

**DOI:** 10.1002/jev2.12085

**Published:** 2021-06-24

**Authors:** Matthew Kang, Vanessa Jordan, Cherie Blenkiron, Lawrence W. Chamley

**Affiliations:** ^1^ Department of Obstetrics and Gynaecology University of Auckland Auckland New Zealand; ^2^ Molecular Medicine and Pathology University of Auckland Auckland New Zealand

**Keywords:** apoptotic body, biodistribution, exosome, extracellular vesicles, microparticle, microvesicle, targeting

## Abstract

In recent years, attention has turned to examining the biodistribution of EVs in recipient animals to bridge between knowledge of EV function in vitro and in vivo. We undertook a systematic review of the literature to summarize the biodistribution of EVs following administration into animals. There were time‐dependent changes in the biodistribution of small‐EVs which were most abundant in the liver. Detection peaked in the liver and kidney in the first hour after administration, while distribution to the lungs and spleen peaked between 2–12 h. Large‐EVs were most abundant in the lungs with localization peaking in the first hour following administration and decreased between 2–12 h. In contrast, large‐EV localization to the liver increased as the levels in the lungs decreased. There was moderate to low localization of large‐EVs to the kidneys while localization to the spleen was typically low. Regardless of the origin or size of the EVs or the recipient species into which the EVs were administered, the biodistribution of the EVs was largely to the liver, lungs, kidneys, and spleen. There was extreme variability in the methodology between studies and we recommend that guidelines should be developed to promote standardization where possible of future EV biodistribution studies.

## 1 INTRODUCTION

Extracellular vesicles (EVs) is a collective term used to describe phospholipid bi‐layered biological packets that are produced and released by all prokaryote and eukaryote cells studied to date. These entities were reported as early as 1946 by Chargaff and West who observed particles derived from platelets from normal plasma (Chargaff & West, 1946).

**FIGURE 1:**
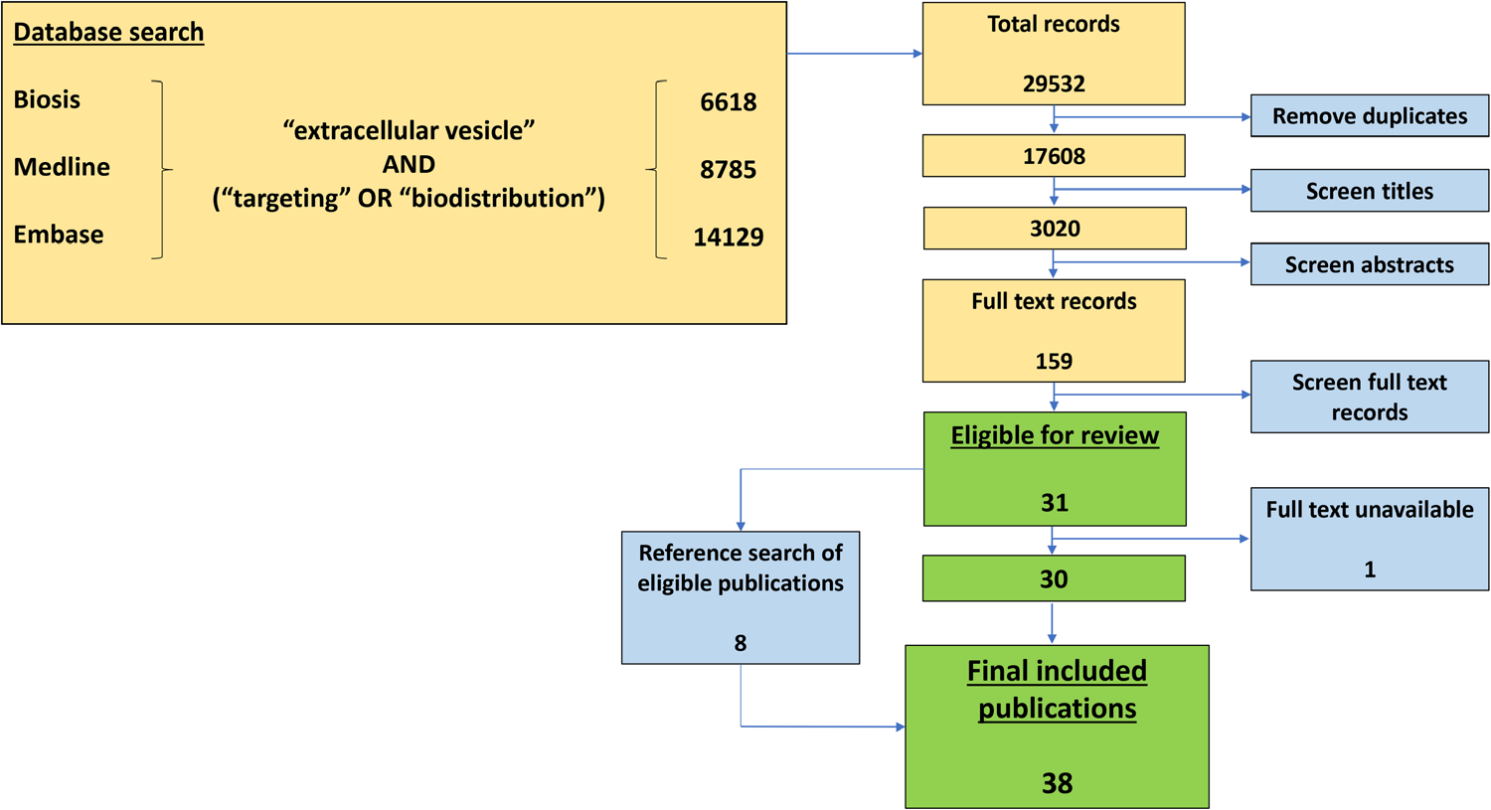
PRISMA‐inspired search of the current literature on EV biodistribution. All publications up to 26th June 2019 were included. Searches were included from three databases: Ovid Biosis, Ovid Medline, and Ovid Embase. Searches were performed for keywords and mesh terms, where available. Keywords used include ‘extracellular vesicle’ AND (‘targeting’ OR ‘biodistribution’), and synonyms

Until recently EVs were thought of as a means of disposing cellular wastes. However, in 1996 Raposo and colleagues showed that B lymphoblastoid cells release EVs carrying MHC class II that could present antigen in vivo inducing antigen‐specific MHC class II‐restricted T cell responses (Raposo et al., 1996). The field of EV research began an exponential increase around 2006 (Witwer & Théry, 2019) when several studies demonstrated that EVs carry biological cargos that can transfer to and alter the behaviour of recipient cells (Ratajczak et al., 2006; Skog et al., 2008; Valadi et al., 2007). Please refer to Figure 1 of https://www.mdpi.com/621550 for a succinct outline of major discoveries in the EV field (Yi et al., 2020).

The nomenclature of EVs in the literature is problematic with multiple disciplines creating different names for EVs. For example, platelet‐derived platelet dust, prostate‐derived prostasomes, cancer cell‐derived oncosomes, dendritic cell‐derived dexasomes, cartilage and bone‐derived matrix vesicles, cardiac progenitor cell and myocyte‐derived cardiosome, etc. (Aalberts et al., 2014; Hargett & Bauer, 2013; Le Pecq, 2005; Tanimura et al., 1983). For greater standardization, typically, EVs are often grouped into three terminologies based on the size of the EVs. Roughly speaking, apoptotic bodies are 800–5000 nm in diameter, microvesicles are between 50–1000 nm in diameter, and nanovesicles (including exosomes) are between 40 and 150 nm in diameter (Crescitelli et al., 2013). Exosomes have been extensively studied and are produced initially as intraluminal vesicles (ILV) in multivesicular bodies (MVB) and are then released from the cell. In contrast, microvesicles are believed to be released by outward blebbing of the cell plasma membrane, while apoptotic bodies are released from cells undergoing apoptosis. Apart from these three, the types of EVs can be dissected even further, as evidenced by Zhang et al. (2018) who showed that exosomes can be fractionated into even smaller sub‐populations called ‘large‐exosomes’, ‘small‐exosomes’, and non‐membranous ‘exomeres’. At the other end of the spectrum, EVs far larger than apoptotic bodies have also been well documented, such as macro‐vesicles that are multi‐nucleated EVs released from the unique multinucleated syncytiotrophoblast of the human placenta. Macrovesicles have a diameter ranging between 20 μm to several hundred micrometres (Tong & Chamley, 2015). Given this wide spectrum of different EV types and nomenclature and confusion over the correct identification of EVs, the ‘Minimal Information for studies of extracellular vesicles (MISEV) 2018′ guideline has suggested the use of two broad terms for EVs based on their size. Small‐EVs are < 100 nm, and large‐EVs are typically > 200 nm (Théry et al., 2018). These MISEV‐derived terminologies will be employed in this review.

Recently the attention has turned to examining the fate of EVs, i.e. their biodistribution upon administration into recipient animals in vivo. Research effort analysing EV biodistribution is a pivotal stepping‐stone in the maturation of the EV field as it highlights the junction between in vitro knowledge and physiological significance. It is especially important for EVs that are designed for therapeutic purposes as it can determine the level of on‐target, as well as off‐target effects of EVs (Yamashita et al., 2018). For example, mesenchymal stem cell (MSC)‐derived small‐EVs have been shown to target preferentially to kidneys in animals with kidney injuries but not control mice (Grange et al., 2014).

Current in vivo studies of EV biodistribution are extremely heterogeneous with widely varying methodologies. Reported studies include wide differences in 1) doses of EVs, 2) routes of administration, 3) recipient animals, 4) organs investigated, 5) timepoints analysed, 6) methods of tracking (such as fluorescent dyes, bioluminescence, radiolabel), and 7) techniques of EV isolation. This diversity creates challenges when trying to compare studies and produce a general understanding of the biodistribution of EVs in vivo.

With these challenges in mind, in this review we aim to summarize the biodistribution of natural EVs that are not ‘modified’ to specifically target cell types or organs. This was done by performing a systemic review of the current literature.

## 2 METHODS

### 2.1 Search strategy

Guided by the PRISMA guidelines, we performed a systemic search of the literature to identify publications that investigated the biodistribution of EVs in vivo. To ensure maximal coverage of the literature, three databases were utilized: Ovid Biosis, Ovid Medline, and Ovid Embase. Search was performed and retrieved on 26th of June 2019 using a broad search category for keywords (‘extracellular vesicle’) AND (‘biodistribution’ OR ‘targeting’) in addition to numerous synonyms for each keyword. The synonyms for extracellular vesicle included (‘microparticle’ OR ‘exosome’ OR ‘microvesicle’ OR ‘nanovesicle’ OR ‘macrovesicle’ OR ‘microparticle’ OR ‘nanoparticle’ OR ‘syncytial nuclear aggregate’ OR ‘shedding vesicle’ OR ‘membrane vesicle’ OR ‘budding vesicle’ OR ‘blebbing vesicle’ OR ‘apoptotic bleb’ OR ‘apoptotic body’ OR ‘extracellular body’ OR ‘exovesicle’). The synonyms for biodistribution included (‘tissue distribution’), while the synonyms for targeting included (‘traffic’ OR ‘docking’ OR ‘recipient’). All records were limited to the English language. The references of the selected records that were eligible for review were screened for additional relevant publications.

### 2.2 Eligibility criteria and search result

A total of 29532 records were retrieved from Biosis (6618), Medline (8785), and Embase (14129) (Figure 1). After removing duplicates, the titles of 17608 records were screened and 14588 records were discarded as the titles did not include keywords, ‘extracellular vesicles’ and ‘biodistribution or targeting’ or its synonyms or that did not present primary data or were conference abstracts. The abstracts of the remaining 3020 studies were screened, and 2681 studies were excluded as they did not relate to EV biodistribution. The full texts of the remaining 159 studies were reviewed in detail. A further 128 studies were excluded as they only contained in vitro analysis or they only included data on modified EVs. All EVs involved in biodistribution studies are unavoidably modified to a certain extent since the labelling process, whether it be fluorescent dyes or proteins, bioluminescence, radiolabel, or labelling for magnetic resonance imaging (MRI), inevitably introduces changes to the EVs. In this review, modified EVs are defined as EVs that have been altered in a way to promote selective organo‐tropic behaviour that would interfere with their natural biodistribution. This left 31 relevant studies. The full text article of one study was not available and therefore excluded from further analysis. An additional eight studies were included in the review after manually screening the reference lists of the 31 relevant studies. In total, 38 studies were eligible for review.

### 2.3 Data collection and quantitative assessment

**FIGURE 2:**
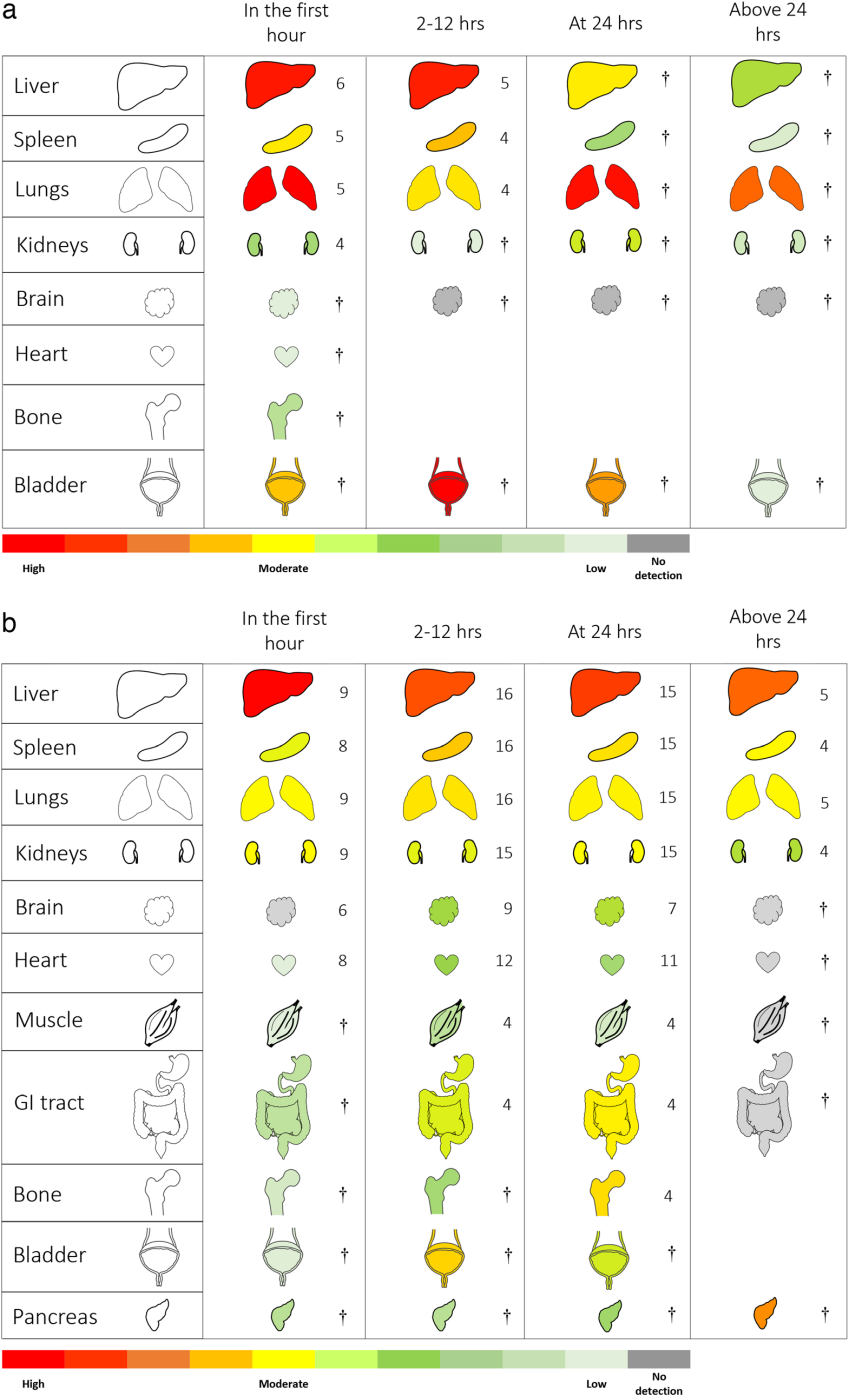
Generalization of small‐EV biodistribution in recipient animals across time after IV administration. **a**. Small‐EV biodistribution collated from seven studies that investigated EV biodistribution through in situ analysis of live whole animals. **b**. Small‐EV biodistribution collated from 36 studies that investigated EV biodistribution through ex vivo analysis of the harvested organs from recipient animals. All organs were represented by at least four studies, indicated by the number beside each organ, except for organs marked with † which indicates representation by three or fewer studies. Abundances of small‐EVs in each organ were calculated as detailed in the methods. Organ images were modified from Smart Servier Medical Art, covered by the Created Commons 3.0 license. https://smart.servier.com/

**FIGURE 3:**
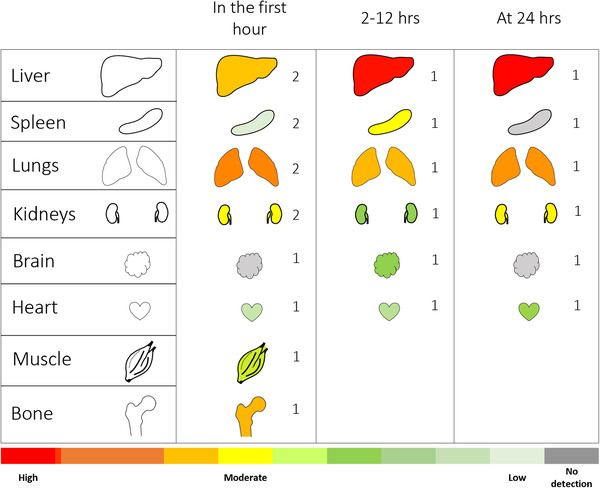
Generalization of large‐EV biodistribution in recipient animals across time after IV administration. Data has been collated from three studies that investigated EV biodistribution through ex vivo analysis of the harvested organs of animals. The representative number of studies for each organ is indicated beside the organ. Abundances of large‐EVs in each organ were calculated as detailed in the methods. Organ images were modified from Smart Servier Medical Art, covered by the Created Commons 3.0 license. https://smart.servier.com/

**FIGURE 4:**
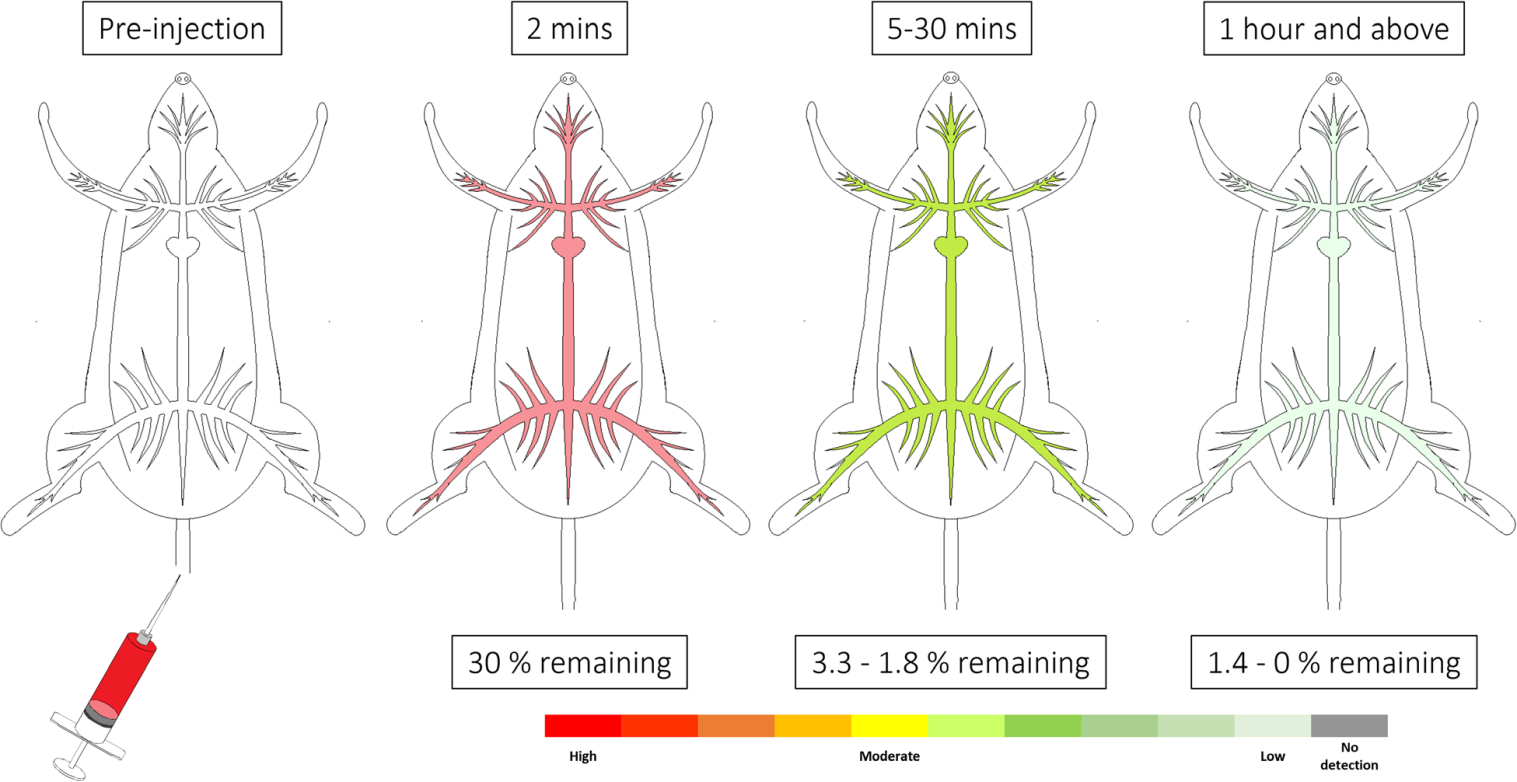
Generalization of small‐EVs detectable in blood circulation across time after IV administration. Data has been collated from six studies that investigated EV levels in blood, without the distinction between serum or plasma. Percentage remaining is represented as a percentage of the injected dose (%ID)

Records eligible for review were thoroughly reviewed and data on organs that were investigated, timepoints, routes of administration, EV tracking methods, and whether they were analyzed by in situ analysis of recipient whole live animals or ex vivo analysis of harvested organs were collected (Supplementary Table 1). Generating a generalization of EV biodistribution required quantitative assessment of EV biodistribution from individual records. Different studies used varying units to illustrate EV biodistribution. Largely, these included expressing EV signals as a signal intensity (e.g., relative fluorescence unit), or as a percentage of total intensity (i.e., intensity combined from all investigated organs), or as a percentage of the initial injected dose. To account for this variance in data representation, data from individual studies were converted to a percentage of total intensity, i.e. signal intensity readings for all the investigated organs were combined to form 100% and signal intensity of individual organs were manually calculated as a percentage of the total. The uniformity of this unit made quantitative comparison between studies possible. This quantification of biodistribution of EVs at each organ was color‐coded according to the author's standard with bright red indicating high levels of EV localization, yellow indicating moderate levels, while pale green indicates low levels of EV localization. Grey indicates no observable EV localization (Figures 2, 3, 4).

## 3 RESULTS

### 3.1 Reporting of EV biodistribution

The 38 studies collectively investigated EV biodistribution in a total of 20 different organs in recipient animals (gastrointestinal (GI) tract, stomach, intestines were collectively considered as a single organ in this review) (Table 1, 2, 3). Recipient animals included different strains of mice and rats. All studies investigated varying selections of organs and no single study investigated all organs. In general, the most frequently studied organs were the liver, spleen, kidneys, and the lungs (reported by a maximum of 33 studies), and as such, a general pattern of EV biodistribution could be determined with greater confidence in these organs. The heart and brain were also commonly studied (reported by a maximum of 26 studies). In contrast, the GI tract, thymus, pancreas, muscle, adipose, bone and bone marrow, lymph nodes, thyroid, bladder, tail, and testicle, were examined less frequently. Additionally, 12 studies examined EV biodistribution to tumours.

The biodistribution of EVs at 23 different timepoints were reported by the 38 studies included in this review. These included the earliest timepoint of 1 min and up to Day 22 post‐EV administration. The most common timepoint was at 24 h, reported by a maximum of 19 studies. For the sake of generalizability, we combined these timepoints into four time‐periods. These time‐periods included ‘In the first hour’, ‘Between 2–12 h’, ‘At 24 h’, and ‘Above 24 h’.

The routes of administration into recipient animals included intravenous (IV, 36 studies), intraperitoneal (IP, three studies), subcutaneous (SC, two studies), oral gavage (one study), and intra‐muscular administration (one study). Given IV administration was the most common route of EV administration reported, this review focuses on the EV biodistribution in recipient animals following IV administration but EV biodistribution following other routes of administration will be mentioned briefly.

Lastly, EV biodistribution following IV administration was investigated through either (or both) in situ analysis of recipient whole live animals (six studies), or ex vivo analysis of organs harvested from recipient animals following euthanasia (34 studies).

### 3.2 Biodistribution of small‐EVs in animals measured by in situ analysis of live intact animals

A total of six studies investigated small‐EV biodistribution via in situ analysis of live whole animals after IV administration of small‐EVs (Table 2). The EVs were fluorescent‐labelled, bioluminescent, or radiolabeled, in two, four, and two studies, respectively. The six studies examined a wide range of time points from 10 min to 12 days post administration of EVs. However, upon removing the data for organs that were represented by three or fewer studies, a general biodistribution pattern of small‐EVs were available for the liver, spleen, lungs, and kidneys in two time‐periods – In the first hour and between 2–12 h.

Using in situ analysis, the liver was the major organ of small‐EV localization, showing peak detection in both the first hour and between 2–12 h (Figure 2a). The lungs showed peak localization of small‐EVs in the first hour but most of this localization was lost between 2–12 h. The spleen was another organ with major localization of small‐EVs, showing moderate levels of detection at both time‐periods. In contrast, the kidneys only showed low levels of small‐EVs in the first hour. There were insufficient reports to generalize on localization of small‐EVs to the kidneys at later time points.

Although there were insufficient data to generalize a pattern for bladder distribution, two studies that did report on the bladder showed opposite findings (Figure 2a) (Royo et al., 2019; Varga et al., 2016). Royo et al. (2019) showed that as early as 15 min after IV administration, small‐EVs could be detected in the bladder, followed by a ∼3‐fold increase by 35 min with a further doubling at 8 h, followed by a decrease thereafter. In contrast, Varga et al. (2016) reported that only low levels of EVs were present in the bladder at 1 h post‐IV administration.

Overall, small‐EV biodistribution interpreted through in situ analysis of live whole animals suggests that following IV administration, small‐EVs typically are localized abundantly in the liver and the lungs, with fewer EVs in the spleen. Whereas the lungs showed a drastic decrease in small‐EVs localization after 1 h, the localization of small‐EVs in the liver was maintained at the same time point. The kidneys showed low levels of EVs in the first hour.

### 3.3 Biodistribution of small‐EVs in animals measured by ex vivo analysis of the harvested organs

A total of 31 studies investigated small‐EV biodistribution in animals following euthanasia and dissection of organs ex vivo after IV administration of small‐EVs (Table 1). The methods of defining EV biodistribution included tracking of fluorescent‐labelled EVs (reported by 26 studies), bioluminescent EVs (three studies), radiolabeled EVs (five studies), miRNA distribution (two studies), liquid chromatography‐mass spectrometry of dopamine concentration packaged into EVs (one study), MRI of gadolinium‐labelled EVs (one study) or super‐paramagnetic iron oxide (SPIO) for MRI and μCT (one study).

The 31 studies examined a wide range of time points ranging from 1 min to 12 days post administration of EVs. After removing the data for organs that were represented by three or fewer studies, the biodistribution of small‐EVs in the liver, spleen, lungs, kidneys, brain, heart, muscle, and GI tract could be generalized across most time‐periods defined as prior. The liver was the major site of small‐EV localization, with peak levels attained in the first hour. The level of small‐EV detection in the liver minimally decreased over the subsequent time‐periods (Figure 2b). Three studies investigated small‐EV distribution to the liver at 48 h and a strong signal remained in the liver at this timepoint (Abello et al., 2019; Antes et al., 2018; Wiklander et al., 2015). A single study reported that at 72 h there was still strong localization of small‐EVs to the liver (Royo et al., 2019). Gangadaran et al. (2017) investigated the two longest timepoints of 6 and 12 days post‐IV administration and showed that small‐EVs were not detectable or minimally detectable in all tested organs at these timepoints.

Despite showing 5 to 6‐fold lower levels of EV localization than the liver, the spleen, kidneys, and lungs demonstrated the next highest levels of EV localization within the first hour after administration. Differences between these three organs were noticeable when comparing the time required to reach peak detection. Whereas the kidneys showed peak levels within the first hour, followed by very gradual reduction thereafter, the spleen and lungs both showed peak levels between 2–12 h.

Distribution to the bladder was only reported by three studies (Goh et al., 2017; Matsumoto et al., 2017; Morishita et al., 2015). There were negligible levels of detection up to 30 min (Matsumoto et al., 2017; Morishita et al., 2015), but increase in detection at 1 h, followed by peak levels at 4 h (Figure 2b) (Morishita et al., 2015). At 24 h, a single study reported moderately low levels of detection (Goh et al., 2017) and no studies reported longer timepoints.

A maximum of 22 studies reported the distribution of small‐EVs to the heart and brain and both organs showed similar patterns of EV localization (Figure 2b). Within the first hour, there were no to minimal EVs localized in these two organs, followed by slight increases in the next two time‐periods. There was insufficient information on brain and heart localization after 24 h.

Distribution of small‐EVs to the GI tract (including stomach and intestines) was reported by 11 studies. There were low levels of small‐EV detection in the GI tract within the first hour after administration (reported by three studies) with gradual increase up to 24 h. Four studies reported on the distribution of small‐EVs to the skeletal muscle and showed minimal localization up to 24 h. Distribution of small‐EVs to the bone (or bone marrow) was reported by seven studies, and only the distribution at 24 h was represented with moderately high levels of localization. Interestingly, if we take into account the two earlier time‐periods which were represented by only two studies each, it appears small‐EV localization in the bone is initially low but peaks to moderately high levels at 24 h. No data was available for above 24 h.

To summarize, the biodistribution of small‐EVs detected in organs ex vivo following IV administration appears to follow three phases of uptake: early (< 1 h), middle (2‐12 h), and late (≥24 h). There was a time‐related increase in the accumulation of small‐EVs in the heart, brain, GI tract, and possibly the bone, potentially indicating late uptake. Detection in the liver, and to a lesser extent kidneys, peaked early with minimal decrease over time. Detection in the lungs and spleen peaked in between these time‐periods.

### 3.4 Biodistribution of small‐EVs in animals administered via non‐intravenous routes

Three studies that reported on IP administration of small‐EVs looked at three timepoints following administration – 24, 48 h, and 22 days. At 24 h, the liver was the major organ of EV distribution (Wiklander et al., 2015). Interestingly, Wiklander et al. (2015) also investigated the distribution to the GI tract and showed similar levels of detection in the GI tract as the liver, followed by the pancreas. In comparison, EV localization to the spleen and lungs were lower, while the localization to the kidneys, heart, and brain were lowest. At 48 h, the spleen and then the liver showed very high levels of EV localization (Alexander et al., 2015; Haney et al., 2019). Alexander et al. (2015) also investigated the distribution to the bone marrow and found moderately high levels of detection. At 22 days, EV localization to the liver was highest, followed by the spleen, while kidneys, lungs, and heart showed low levels of localization (Haney et al., 2019).

A single study performed ex vivo analysis of the biodistribution of small‐EVs at 24 h following subcutaneous (SC) administration and demonstrated that the GI tract was the major site of EV localization, followed by the liver (earlier time points were not reported) (Wiklander et al., 2015). The lungs and pancreas showed moderately high levels of EVs, while the spleen, kidneys, heart, brain, and skeletal muscle contained low levels of EVs. Another study examining EV biodistribution 72 h after SC administration demonstrated that EVs were predominantly localized in the thyroid (Royo et al., 2019). Although substantially lower than the detection at the thyroid, the kidneys appeared to show the second highest EV localization, followed by the liver. The lungs and spleen contained lower levels of EVs, followed by the intestines, heart, and brain (Royo et al., 2019).

Only one study examined the biodistribution of small‐EVs following intramuscular administration and found that the EVs were predominantly localized to the GI tract, closely followed by the liver (Jalabert et al., 2016). The lungs and the pancreas contained moderate levels of EVs, while the spleen, kidneys, heart, brain and skeletal muscle contained few or no EVs.

Finally, one study investigated the biodistribution of bovine milk small‐EVs in female BALB/c mice following administration via oral gavage (OG) and found strong localization to the intestines followed by the liver at 24 h (Manca et al., 2018). At 48 h, no signal was detectable.

### 3.5 Biodistribution of large‐EVs in animals measured by ex vivo analysis of the harvested organs

Only three studies investigated large‐EV biodistribution in animals following IV administration (Table 3). As the number of studies investigating large‐EV biodistribution was very limited, the following summary for large‐EV biodistribution should be viewed with caution. In all of these, the biodistribution was quantified via ex vivo analysis of target organs post‐euthanasia. Two of these three studies tracked the fluorescently labelled large‐EVs while one tracked radiolabeled EVs.

The three studies examined time points ranging from 2 min to 24 h post administration of EVs (Figure 3). Early after IV administration, the large‐EVs rapidly localized to the lungs peaking in the first hour and thereafter levels declined between 2–12 h. In contrast, large‐EVs began accumulating in the liver increasingly from the first hour peaking at 24 h. There appeared to be moderate to low levels of large‐EVs localized to the kidneys across time, while the brain and heart showed low to no localization of large‐EVs across time. In contrast, there were moderately low to no levels of large‐EVs localized to the spleen across the three time‐periods up to 24 h.

### 3.6 Biodistribution of large and small‐EVs to tumours

We found 12 studies that specifically examined the biodistribution of EVs in mice that had been grafted with tumours, of which 11 studies investigated small‐EVs and one investigated large‐EVs after IV administration.

Within the first hour after IV administration of small‐EVs, only a single study was included. Between 2–12 h, six studies were included, and at 24 h, six studies were included. Finally, above 24 h, again only a single study was included. In general, within the first hour, there was no detection of small‐EVs in the tumour (Jung et al., 2018). Between 2–12 h and at 24 h, moderate levels of EVs were detected in tumours (Abello et al., 2019; Gao et al., 2018; Goh et al., 2017; Kim et al., 2017; Kooijmans et al., 2016; Lee et al., 2018; Li et al., 2018; Smyth et al., 2015; Wiklander et al., 2015). Finally, above 24 h, a moderately low detection in the tumour was observed (Abello et al., 2019).

Only a single study examined large‐EV localization to tumours (Zhang et al., 2017) and reported moderate levels of large‐EVs localized at the tumours between 2–12 h. Other time‐periods were not reported.

### 3.7 Circulating levels of EVs following IV administration

15 studies investigated the presence of small‐EVs in the circulation following IV administration, but of these only six studies reported comparable parameters (Imai et al., 2015; Lai et al., 2014; Matsumoto et al., 2017; Morishita et al., 2015; Takahashi et al., 2013; Zhang et al., 2018). To enable comparison, we combined the data into three time‐periods; ‘2 min’, ‘5 to 30 min’, and ‘1 h and above’ post injection. All six studies reported the EVs remaining in circulation as a percentage of the injected dose (%ID).

In general, in the first 2 min following IV administration, 30% of administered small‐EVs remained in the circulation and this dropped to 1.8 to 3.3% between 5–30 min (Figure 4). From 1 h onwards, 1.4 to 0% of the injected small‐EVs remained in the circulation. Three studies reported that ∼95% of the administered small‐EVs were removed from the circulation within 5 min (Matsumoto et al., 2017; Morishita et al., 2015; Takahashi et al., 2013). Half‐life pharmacokinetic data, available from four studies, showed T1/2α (distribution phase) ranging between 1.5‐19.9 min and T1/2β (elimination phase) between 34.6‐184.5 min (Table 4) (Lai et al., 2014; Matsumoto et al., 2017; Morishita et al., 2015; Takahashi et al., 2013). Area under the curve data reported by four studies ranged between 0.7‐3.2 % dose h/ml while mean residence time ranged between 0.5‐0.847 h (Imai et al., 2015; Matsumoto et al., 2017; Morishita et al., 2015; Takahashi et al., 2013).

**TABLE 1:** List of experimental parameters used by studies that investigated small‐EV biodistribution by ex vivo analysis

Reference	Source of EV	Administration route	Dose of EV	Recipient animal	Organs investigated	Timepoints investigated for biodistribution
(Abello et al., 2019)	Human MSC	IV	Gadolinium: 0.015 mmol/kg, DiR: 5 mg/kg in 100 μl DMEM	Immunodeficient NU/NU nude mice ‐ bearing K7M2 tumour	Liver, kidney, spleen, lung, heart, bone, tumour	24, 48 h
(Alexander et al., 2015)	miR155‐/‐ C57BL/6 mice‐derived bone marrow‐DC	IP	∼10^9^ particles	miR155‐/‐ C57BL/6 mice	Liver, spleen, bone marrow	48 h
(Antes et al., 2018)	Cardiosphere‐derived cell	IV	10^9^ particles in 1 ml PBS	Female Wistar Kyoto rats ‐ with I/R injury, Female Wistar Kyoto rats ‐ no injury	Heart, liver, lung, spleen, kidney	48 h
(Bala et al., 2015)	M.12.4.1 cell	IV		C57BL6J mice ‐ with miR155 KO	Liver, adipose, lung, muscle, kidney, brain, thymus, heart	10, 40 min
(Chen et al., 2018)	AML12 hepatocyte cell	IV	40 μg	Swiss Webster mice	Liver, lung, heart, spleen, kidney	4 h
(Gangadaran et al., 2017)	Cal62 cell, MDA231 cell	IV	25 μg protein weight	Female BALB/c nude mice	Lung, liver, spleen, kidney	3 h, 6, 12 days
(Gao et al., 2018)	C2C12 cell, Hepa 1–6 cell, Human serum	IV	30 μg protein weight	C57BL/6 mice, C57BL/6 mice ‐ bearing HCC tumour, Immunodeficient nude mice	Liver, spleen, kidney, lung, heart, brain, intestine, muscle, tumour	2 h
(Goh et al., 2017)	U937 cell	IV	40 μg/100 μl	White BALB/c mice ‐ bearing CT26 tumour	Lung, heart, spleen, brain, colon, liver, bladder, kidney, tumour	24 h
(Grange et al., 2014)	MSC	IV	200 μg of DiD labeled EV	Male CD1 nude mice ‐ healthy control, Male CD1 nude mice ‐ acute kidney injury	Kidney, spleen, liver, lung	5, 24 h
(Haney et al., 2019)	IC21 cell	IP	2 × 10^11^ in 200 μl saline	LINCL mice ‐ having a mutation of CLN2 gene was used as a model of Batten disease	Liver, lung, spleen, kidney, brain	22 days
(Jalabert et al., 2016)	HEK293 cell, Muscle from mice fed Standard chow diet, Muscle from mice fed High palmitate diet	IV, IM	IV: 2 × 10^11^ particles in 200 μl PBS, IM: 2 × 10^11^ particles in 50 μl PBS	Female NMRI mice	GI tract, liver, lung, pancreas, kidney, spleen, quadricep, heart, brain	24 h
(Jung et al., 2018)	MDA‐MB231 cell, Hypoxic grown‐MDA‐MB231 cell	IV	100 μg	Female BALB/c nu/nu mice, Female BALB/c nu/nu mice ‐ bearing MDA‐MB231 tumour	Brain, lung, heart, liver, spleen, kidney, intestine, tumour	60 min
(Kim et al., 2017)	HEK293 cell, SKOV3 cell	IV	10 mg/kg	Female BALB/c nude mice ‐ bearing SKOV3 tumour	Liver, lung, heart, kidney, spleen, tumour	24 h
(Kooijmans et al., 2016)	Neuro2A cell	IV	6 μg in 100 μl PBS	Female Crl:NU‐Foxn1nu mice (immunocompromised) ‐ bearing A431 tumour	Liver, spleen, kidney, lung, brain, tumour	4 h
(Lai et al., 2014)	HEK293T cell	IV		Athymic nude mice	Spleen, liver, lung, kidney, brain, heart, muscle	30, 60 min, 2, 6 h
(Lee et al., 2018)	MCF‐7 cell, MDA‐MB231 cell	IV	10 μg protein weight in 0.1 ml TBS	Female athymic mice ‐ bearing MCF‐7 tumour, Female athymic mice ‐ bearing MDA‐MB231 tumour	Liver, lung, spleen, kidney, small intestine, large intestine, muscle, tumour	4, 24 h
(Li et al., 2018)	LIM1215 cell	IV	DiR dose of 50 μg/kg	BALB/c nude ‐ bearing LIM1215 xenograft	Heart, liver, spleen, lung, kidney, tumour	12 h
(Manca et al., 2018)	Bovine milk, Transgenic pig	IV, OG	1 × 10^10^/g, 1 × 10^11^/g, 1 × 10^12^/g	Female BALB/c mice, Male BALB/c mice, Macrophage‐depleted female BALB/c mice	Liver, spleen, kidney, heart, lung, brain, intestine	3, 6, 24 h
(Matsumoto et al., 2017)	B16BL6 cell	IV	4 μg (protein weight) per mouse	Male BALB/c mice ‐ pretreated with PBS, Male BALB/c mice ‐ pretreated with PC liposome, Male BALB/c mice ‐ pretreated with PS liposome	Brain, heart, lung, spleen, liver, stomach, intestine, kidney, bladder, tail, adipose, bone	5 min
(Morishita et al., 2015)	B16BL6 cell	IV	4 μg of exosome protein/mouse	Male BALB/c mice	Brain, heart, lung, spleen, liver, stomach, intestine, kidney, bladder, tail, adipose, bone	1, 5, 10, 30, 60 min, 4 h
(Nordin et al., 2015)	UC‐purified EV, UF‐LC‐purified EV	IV		BALB/c mice	Lung, liver, spleen, kidney	24 h
(Qu et al., 2018)	Blood	IV	Dopamine dose of 18 mg/kg, 10 mg/kg	Kunming mice	Heart, liver, spleen, lung, kidney, brain	1, 4, 6, 8, 12 h
(Royo et al., 2019)	MLP29 cell	IV, SC	IV: 120 ng of protein weight in 150 μl, SC: 40 ng of protein weight in 30 μl	Male BALB/cJRj mice	Thyroid, lung, heart, kidney, spleen, testicle, liver, small intestine, brain	72 h
(Smyth et al., 2015)	4T1 cell, PC3 cell, MCF‐7 cell	IV	60 μg in 200 μl PBS, 30‐32 μg for radiolabeled exosomes	BALB/c mice ‐ bearing 4T1 tumour, Nude NU/J mice ‐ bearing PC3 tumour, Nude NU/J mice ‐ no tumour control, BALB/c mice ‐ healthy immune system, NOD.CB17‐Prkdcscid/J ‐ impaired innate immunity + impaired complement activity, Nude NU/J ‐ lack of adaptive immunity	Heart, lung, liver, spleen, kidney, stomach, intestine, bone, muscle, tumour	2, 24 h
(Tamura et al., 2017)	MSC	IV	10 μg (protein) in 100 μl PBS	C57B6 mice ‐ with liver damage	Lung, heart, liver, spleen, intestine, kidney	4, 24 h
(Takahashi et al., 2013)	B16‐BL6 cell, B16‐BL6 cell transfected to express Gluc‐lactadherin	IV	5 μg exosome protein/mouse	Male BALB/c mice, C57BL/6 mice	Liver, kidney, spleen, lung, stomach, heart, brain, intestine	30 min, 4 h
(Tong et al., 2017)	First trimester placenta	IV	100 μg protein weight	Female CD1 mice ‐ pregnant	Brain, thymus, heart, lung, liver, spleen, pancreas, kidney, feto‐placental unit, skeletal muscle	30 min, 24 h
(Viñas et al., 2018)	Endothelial colony forming cell	IV	20 μg in 100 μl PBS	Male FVB mice ‐ I/R injury	Kidneys, liver, heart, lung, spleen	3 min, 4, 24 h
(Wen et al., 2016)	EO771 cell, 4T1 cell, 67NR cell	IV	EO771: 20 μg (equates to 1.6 × 10^11^ particles), 4T1: 20 μg (equates to 1.2 × 10^11^), 67NR: 20 μg (equates to 1.2 × 10^11^)	C57BL/6 mice, BALB/c mice	Liver, spleen, kidney, heart, lung, bone marrow	24 h
(Wiklander et al., 2015)	HEK293T cell, C2C12 cell, B6B16 cell, DC	IV, IP, SC	1 × 10^10^ p/g, 0.25 × 10^10^ p/g, 1 × 10^10^ p/g, 1.5 × 10^10^ p/g	Female NMRI mice, C57BL/6 mice ‐ bearing B16‐F10 tumour, C57BL/6 mice	Lung, liver, spleen, pancreas, GI tract, brain, heart, kidney, quadricep, tumour	5, 30, 60 min, 3, 24, 48 h
(Zhang et al., 2018)	B16‐F10 cell	IV	10 μg of nanoparticles	Female naïve C57BL/6 mice	Liver, lung, lymph node, spleen, bone, kidney, brain, heart	24 h
(Zhang et al., 2018)	MCF‐7 cell, MDA‐MB231 cell, HS578T cell	IV	20 μg (protein weight) in 100 μl PBS	Female BALB/c nude mice	Brain, liver, spleen, lung, kidney	24 h
(Wang et al., 2017)	DC	IV	50 μg	Female BALB/c nude ‐ bearing MDA‐MB231 tumour	Liver, spleen, lung, lymph node, heart, brain, tumour	4.5 h

Abbreviations: IV, intravenous; IP, intraperitoneal; SC, subcutaneous; IM, intramuscular; OG, oral gavage; Gluc, Gaussia Luciferase; MSC, mesenchymal stem cell; DC, dendritic cell; I/R, ischaemia/reperfusion; UC, ultracentrifugation; UF‐LC, ultrafiltration‐liquid chromatography; PC, phosphatidylcholine; PS, phosphatidylserine.

**TABLE 2:** List of experimental parameters used by studies that investigated small‐EV biodistribution by in situ analysis

Reference	Source of EV	Administration route	Dose of EV	Recipient animal	Organs investigated	Timepoints investigated for biodistribution
(Gangadaran et al., 2017)	Cal62 cell, MDA231 cell	IV	25 μg protein weight	Female BALB/c nude mice	Lung, liver, spleen regions	10, 30, 60 min, 3, 24, 48, 72 h, 6, 12 days
(Imai et al., 2015)	Gluc‐LA‐transfected B16BL6 cell	IV	5 μg of exosomal protein weight	BALB/c mice ‐ clodronate liposome pretreatment		10, 30, 60 min, 4 h
(Lai et al., 2014)	HEK293T cell	IV	GlucB‐EV at bolus of 100 μg	Athymic nude mice	Whole animal	30 min
(Royo et al., 2019)	MLP29 cell	IV	120 ng of protein weight in 150 μl	Male BALB/cJRj mice	Whole animal: VOI: bladder, liver, thyroid, lung, kidneys, brain	15, 35 min, 8, 24, 48, 72 h
(Takahashi et al., 2013)	Gluc‐lactadherin‐expressing B16BL6 cell	IV	5 μg exosome protein/shot	White BALB/C mice	Whole animal	10, 30, 60 min, 4 h
(Varga et al., 2016)	Erythrocyte	IV	15±2 MBq of ^99m^Tc‐labeled erythrocyte EV in 200 μl	Male BALB/c mice	Whole animal: VOI: heart, lung, kidneys, bladder, liver, spleen, bone	1 h

Abbreviations: IV, intravenous; Gluc, Gaussia Luciferase; GlucB, membrane‐bound Gluc, LA, lactadherin; VOI, volume‐of‐interest.

**TABLE 3:** List of experimental parameters used by studies that investigated large‐EV biodistribution by ex vivo analysis

Reference	Source of EV	Administration route	Dose of EV	Recipient animal	Organs investigated	Timepoints investigated for biodistribution
(Tong et al., 2017)	First trimester placenta	IV	300 μg	Female CD1 mice ‐ non‐pregnant, Female CD1 mice ‐ pregnant	Brain, thymus, heart, lung, liver, spleen, pancreas, kidney, uterus/placenta, skeletal muscle	2, 30 min, 24 h
(Willekens et al., 2005)	Wistar rat blood	IV	480 μl of ^51^Cr‐labeled vesicles	Male Wistar rat	Liver, bone, skin, muscle, spleen, kidney, lung	30 min
(Zhang et al., 2017)	Macrophage	IV	100 μl, 1 mg/ml	Female BALB/c nude mice ‐ bearing Hela tumour	Liver, spleen, lung, kidney, heart, brain, tumour	4 h

Abbreviation: IV, intravenous.

**TABLE 4:** Pharmacokinetic parameters retrieved from a maximum of four studies

T1/2α (minutes)	T1/2β (minutes)	AUC (% dose h/ml)	MRT (hours)
1.5 ‐ 19.9	34.6 ‐ 184.5	0.7 ‐ 3.2	0.5 ‐ 0.847

T1/2α, half‐life of distribution phase; T1/2β, half‐life of elimination phase; AUC, area under the curve; MRT, mean residence time of EVs

Only a single study reported the fate of large‐EVs in the circulation following IV administration (Willekens et al., 2005). Willekens et al. (2005) reported that only 30% of the administered large‐EVs remained in the blood after the first 2 min, reducing to 9% of ID at 30 min.

## 4 DISCUSSION

### 4.1 Comparison of the biodistribution of small‐EVs analyzed by in situ and ex vivo analysis

Powerful comparison between in situ and ex vivo analysis of biodistribution was limited largely because fewer organs were analyzed in situ compared to ex vivo. Moreover, a maximum of four time‐periods were available for ex vivo analysis of organ biodistribution, while only the two earlier time‐periods were available for in situ analysis (Figure 2a & b). This made it impossible to compare the biodistribution patterns from 24 h and above following IV administration.

Small‐EV localization at the liver and spleen were similar between the two imaging systems in the first two time‐periods up to 12 h. However, upon taking into account the less represented data for the liver and spleen analyzed in situ at 24 h and above, it appeared that the detection of EVs at these two later time‐periods were substantially less compared to the detection seen through ex vivo analysis, which showed minimal loss across all time‐periods (Figure 2a & b). If this observation is true, the discrepancy between the two imaging systems could potentially be explained by depth limitations associated with optical imaging of animals, a common method for in situ analysis (Gangadaran et al., 2018; Wiklander et al., 2015). Ex vivo analysis can avoid this problem and acquire stronger detection of labelled‐EV signals.

Similarly, including the less represented data for bladder distribution for both in situ and ex vivo analysis appeared to show a similar pattern of distribution in this organ. In both cases, peak detection was observed between 2–12 h time‐period, which decreased thereafter. These dynamics of small‐EV distribution to the bladder occurred while kidneys showed low levels of distribution, and if this observation is true, it might indicate that the bulk of the small‐EVs are cleared through the kidneys into the urine without being taken up and retained in the cells of the kidneys.

The major difference in biodistribution between the two imaging systems was seen in the lungs, whereby in situ analysis reported very high EV localization in the first hour while ex vivo reported only moderate levels. The reason for this discrepancy might be an artefact of the meta‐analysis process.

### 4.2 Distribution of EVs to the lungs

The administration of EVs into recipient animals is most commonly performed by IV administration into the tail vein of either mice or less commonly rats. Following tail vein injection, EVs travel via the heart to the lungs where they encounter the first capillary bed. Given that the diameter of micro‐capillaries in mouse lung can be as small as 1–2 μm (Hayashi et al., 2018; Nguyen et al., 2019; Townsley, 2012), the physical size of EVs may contribute to the localization of EVs, especially large‐EVs to the lungs. That there is an initial high distribution of large‐EVs to the lungs followed by redistribution to other organs, physical ‘entrapment’ in the pulmonary capillaries may explain the initial localization in the lungs. However, given that mice typically recirculate their blood volume nine times each minute and several studies of both large‐ and small‐EVs showed persistence of EVs in the lungs for up to 24 h, physical entrapment seems unlikely to be the sole mechanism by which EVs remain localized to the lungs (Linden et al., 2012).

It is likely that cellular uptake is also occurring in pulmonary cells. Indeed, small‐EVs from breast cancer MDA‐MB231 sub‐cell line (4175) localized to the lungs following IV (retro‐orbital) administration with uptake by S100A4^+^ fibroblasts and surfactant protein C^+^ epithelial cells. The localization of the EVs was dependent on integrin α6β4 and α6β1 expression on small‐EVs (Hoshino et al., 2015). In another study, melanoma B16BL6 cell‐derived small‐EVs were localized to the lungs 10 min after injection but treating the EVs with proteinase K reduced vesicular integrin α6β1 and diminished distribution to the lungs (Charoenviriyakul et al., 2018). This reinforced the finding that surface expression of integrin α6β1 might potentially be related to lung distribution of EVs.

Interestingly, inconsistent findings were noticed between two studies that used the same cell line‐derived small‐EVs (Faruqu et al., 2019; Peinado et al., 2012). Whilst Peinado et al. (2012) showed B16F10 cell‐derived small‐EVs in the lung sections of mice at all timepoints (5 min and 24 h after IV administration), Faruqu et al. (2019) showed that B16F10 cell‐derived small‐EVs did not localize to the lungs in appreciable amounts at all timepoints (1, 4 and 24 h), with the majority of small‐EVs detected in the liver and spleen. Although the precise reason behind this is not known, it could potentially be attributed to two differences between the studies. Firstly, the different imaging modalities used to analyze EV detection – one study counted the gamma released from [^111^In]‐radiolabelled small‐EV from the harvested organs of mice after small‐EV administration (Faruqu et al., 2019) while the other study detected for co‐localization of PKH67 fluorescently labelled small‐EVs in lung sections using confocal microscopy (Peinado et al., 2012). Secondly, the dose could have also made a difference. However, the units of dose provided by each study were incompatible and a comparison was not possible. This inconsistency in biodistribution pattern highlights the potential issue in the current field – the extreme variability in the methodology may create different interpretations of biodistribution.

### 4.3 Distribution of EVs to the liver

The liver is the largest organ in the body but this large size and high blood flow (945 ± 242 ml/min) in mice alone cannot explain the high distribution (Gjedde & Gjeode, 1980; Hui et al., 1994).

The liver is a major clearance organ for many substances containing a population of resident macrophages called Kupffer cells, which in mice can be identified by the F4/80 marker. Several studies suggest that EVs are taken up predominantly by Kupffer cells although both small‐ and large‐EVs are also taken up by hepatocytes and other cells in the liver (Bala et al., 2015; Imai et al., 2015; Tamura et al., 2017; Wang et al., 2018; Willekens et al., 2005).

Current evidence suggests that scavenger receptors, which are particularly abundant on Kupffer cells, are the key players involved in EV uptake. Scavenger receptors can bind to numerous ligands to promote removal of non‐self or altered‐self targets including phosphatidyl serine (PS) which is enriched in the membrane of many EVs (Llorente et al., 2013; Prabhudas et al., 2017). Several groups have elegantly demonstrated the role of scavenger receptors by pre‐injecting animals with blocking ligands, including negatively charged PS‐rich liposomes or poly‐inosinic acid (poly‐I), prior to IV administration of large‐EVs. In those experiments, EVs showed slower clearance from the blood and significantly reduced liver localization. In contrast, phosphatidylcholine‐rich liposome, which are not negatively charged had no effect on EV localization to the liver (Matsumoto et al., 2017; Willekens et al., 2005). The role of the negative charge in this localization is reinforced by the finding that negatively, but not positively charged non‐biological nanoparticles are rapidly taken up by Kupffer cells in the liver (Cheng et al., 2012).

As for lung, the presence of specific integrins on the surface of EVs are also involved in targeting EVs to the liver (Murphy et al., 2019). This has been elegantly shown by Hoshino et al. (2015), who performed proteomic analysis of 28 organ‐specific metastatic cell line‐derived small‐EVs. Integrins were the largest group of highly abundant adhesion molecules and liver tropism was conferred by the expression of integrin αvβ5 on small‐EVs, which particularly associated with Kupffer cells (90% of small‐EV positive cells).

Interestingly, when all macrophages were depleted by pre‐injecting clodronate liposomes into mice, IV administered B16BL6 cell‐derived small‐EVs still distributed to the liver, which suggested that non‐macrophage cells are also involved in the uptake of small‐EVs in the liver (Imai et al., 2015). Apart from the uptake by Kupffer cells, a recent study has shown that liver sinusoidal endothelial cells (LSECs) can also take up hepatic stellate cell (HSC)‐derived small‐EVs (Wan et al., 2019).

### 4.4 The distribution of EVs to the spleen

The spleen is a lymphoid organ involved in mounting immune responses to blood‐borne antigens and was a site of considerable localization of small‐EVs and to a lesser extent large‐EVs (Batista et al., 2016).

Following entry of small‐EVs into the spleen, metallophillic macrophages present in the marginal zone are the first cells exposed to EVs. These macrophages are characterized by CD169 (SIGLEC‐1) expression (Grabowska et al., 2018). Despite the low affinity of CD169 for sialic acid, they are capable of strongly binding to heavily sialylated multimeric structures, such as some small‐EVs (Saunderson et al., 2014). This has been demonstrated in vivo with B cell‐derived small‐EVs which are enriched with α2,3‐linked sialic acids (Saunderson et al., 2014). Following IV or SC administration, B‐cell‐derived small‐EVs localized with marginal zone macrophages that expressed CD169. Sialidase treatment, which cleaves the terminal sialic acid residues, of small‐EVs resulted in the failure to localize to splenic marginal zone macrophages.

Another mechanism of targeting small‐EVs to the spleen requires the presence of CC chemokine receptor 7 (CCR7) on the EVs. CCR7 is highly expressed on mature dendritic cells and the small‐EVs they produce. Down‐regulation of CCR7 in dendritic cells resulted in small‐EVs with reduced homing to the spleen (Wei et al., 2017).

Different subpopulations of leukocytes may be involved in the uptake of small‐EVs in the spleen. One study investigated the uptake efficiency of tumour‐derived small‐EVs by a panel of leukocyte subpopulations in the spleen and revealed that CD4+, CD8+, and sIgM+ lymphocytes showed uptake of small‐EVs in vitro but there was much greater uptake of small‐EVs by CD11b+ macrophages and CD11c+ DCs (Zech et al., 2012).

Although there were limited numbers of studies that investigated the distribution of large‐EVs to the spleen, generally this was lower than the localization of small‐EVs to the spleen.

Interestingly, transcardial perfusion of animals (to eliminate free EVs in blood) prior to imaging the organ caused a substantial decrease in the localization of EVs to the spleen (Lai et al., 2014). The authors argued that the EVs are not efficiently taken up into splenic cells but that the organ serves as a transitory reservoir for EVs when they are administered in excess, i.e. that EVs in excess will saturate the liver macrophages and ‘spill‐over’ into the splenic vasculature. However, Wiklander et al. (2015) reported that increasing the dose of small‐EVs administered IV did not increase the distribution of EVs to the spleen.

### 4.5 Distribution of EVs to the kidneys

It is clear that small‐EVs administered systemically can reach the kidneys and subsequently the urine (Cheng et al., 2012). Consistently, although not as prominent as the detection of EVs in the liver or lungs, the generalized summary in this review outlined moderate levels of small‐EVs detected in the kidneys across time. Likewise, moderate levels of large‐EVs were detected in the kidneys across time.

Although the mechanisms of action of EV uptake by cells in the kidneys are yet to be fully understood, the size constraints of EVs at the glomerular filtration barrier (4.5‐5 nm in diameter) would suggest that passive diffusion of intact EVs to the urine is unlikely (Longmire et al., 2008).

Unfortunately, it was not possible to generalize the changes in EV biodistribution to the bladder in this review because there were insufficient number of studies investigating the bladder.

### 4.6 Distribution of EVs to the tumour

Generally speaking, small‐EVs were detectable in tumours between 2–12 h and at 24 h after administration. Large‐EVs were detectable in tumours only between 2–12 h post injection. Tumours have leakier vasculature than healthy blood vessels and impaired lymphatic drainage (Bae & Park, 2011; Walker et al., 2019) which is believed to allow easier passage of nanoparticles into the interstitium and cause high retention within the tumour tissue. As EVs can be in the size range of liposomes/nanoparticles, it is possible that accumulation of EVs in tumours could also occur through the same process (Wiklander et al., 2015).

However, there was some variation in the tumour distribution of EVs between studies. At one end of the spectrum, small‐EVs could not be detected in tumour in three studies across several timepoints, including 1, 2, 4, and 24 h (Jung et al., 2018; Kooijmans et al., 2016; Smyth et al., 2015). At the other end of the spectrum, heavy accumulation of small‐EVs was detected in tumours 24 h after IV administration (Kim et al., 2017).

Unfortunately, a discernible correlation of experimental parameters between studies could not be made to explain the apparent variance in tumour distribution observed between studies. For example, the three studies that reported no detection in tumours used varying doses (6 μg, 60 μg, 100 μg) and covered different timepoints (1, 2, 4, 24 h) (Jung et al., 2018; Kooijmans et al., 2016; Smyth et al., 2015). Different recipient mouse strains also did not make a difference in tumour distribution of EVs. This was clearly demonstrated by Smyth et al. (2015) who performed a side‐by‐side comparison of 4T1 cell‐derived small‐EV distribution to the tumours in three mouse strains, including BALB/c (immunocompetent), nude NU/J (lacking adaptive immunity), and NOD/CB17‐Prkdcscid/J (lacking innate immunity and complement system), and found no tumour distribution in all three mice strains.

One experimental parameter that does appear to make a difference in tumour distribution is the route of administration of EVs. Direct injection of EVs into a target tumour or in proximity to the tumour resulted in higher levels of EVs retained in the tumour than the EVs administered IV (Jaiswal et al., 2013; Smyth et al., 2015). In another study (not included in this review), EVs were made to encapsulate oncolytic viruses (EV‐virus), which can exhibit natural tumour‐selective tropism. Comparison of the tumour distribution of IV and IP administered EV‐virus in C57BL/6 mice showed that IV administration resulted in a strong localization of the EVs to the tumour whereas, IP administration resulted in minimal tumour distribution at 24 h. The authors speculate that this may be due to the specific peritoneal environment modifying the formulation of the EVs following IP administration, essentially interfering with the homing capability of EVs (Garofalo et al., 2018).

Another method of improving tumour targeting by EVs, particularly of interest for the development of EV therapeutics is by modifying the surface ligands of EVs. For example, dendritic cell‐derived large‐EVs were engineered to carry AS1411, a DNA aptamer that binds to nucleolin overexpressed on breast cancer cells. Upon IV administration, there was stronger tumour accumulation of modified large‐EVs compared to unmodified large‐EVs (Wang et al., 2017). Tumour targeting by dendritic cell‐derived EVs was also increased by engineering the cells to express Lamp2b fused to an iRGD peptide sequence that is specific for integrin αv, integrins highly expressed on tumour cells (Tian et al., 2014).

### 4.7 Clearance of EVs from the blood

It is clear that a large portion of EVs, regardless of whether they are small‐ or large‐EVs and regardless of the cell of origin, are rapidly removed from the circulation of animals to which the EVs are administered. Generally speaking, this clearance was effective within minutes of administration of EVs. This could be explained by a two‐phase exponential decay model whereby EVs in circulation are initially and rapidly distributed to organs (distribution phase) characterized by a short half‐life (T1/2α), followed by a gradual elimination of EVs via the liver and kidneys (elimination phase) characterized by a longer half‐life (T1/2β) (Lai et al., 2014; Takahashi et al., 2013).

### 4.8 Selecting an appropriate animal model

Choosing an appropriate animal model is an important consideration that may influence the biodistribution pattern of EVs. There are several studies in the literature that have directly investigated the side‐by‐side comparison of different animal models. One study compared the biodistribution of 4T1 cell‐derived small‐EVs in three different mouse models with different immune systems: BALB/c (healthy), Nude NU/J mice (lack of adaptive immunity), and NOD.CB17‐Prkdcscid/J mice (impaired innate immunity + impaired complement activity). They found that only the mice with impaired innate immune and complement systems showed slower uptake of small‐EVs by reticuloendothelial system (RES), implicating the significance of innate immunity in the clearance and biodistribution of small‐EVs (Smyth et al., 2015). The sex of the animal also appears to influence EV biodistribution as has been demonstrated by the reduced fluorescent signals of bovine milk small‐EVs in male BALB/c compared to female BALB/c mice (Manca et al., 2018). More studies are warranted to determine whether this finding is specific to milk‐derived EVs or universally affects EVs. Pregnancy can also influence EV biodistribution. Tong et al. (2017) demonstrated that the biodistribution pattern of large‐EVs is different between pregnant and non‐pregnant CD1 mice, with more large‐EVs detected in the lungs of pregnant mice compared to non‐pregnant mice at both 30 min and 24 h. Interestingly, the presence of tumour in mice did not significantly alter the biodistribution of administered EVs to other main organs, including liver, spleen, and kidneys (Jung et al., 2018; Smyth et al., 2015; Wiklander et al., 2015).

### 4.9 Selecting the appropriate imaging modality

The biodistribution of EVs can be tracked through diverse imaging modalities. These typically include optical imaging and nuclear imaging. Optical imaging utilizes the absorption and emission of photons in the wavelength of visible and infrared light. Nuclear imaging utilizes the detection of radionuclides through their emission of gamma rays or positrons. In a simplistic view, the optimal method would be one that offers high sensitivity, high spatial and temporal resolution, high signal‐to‐noise ratio, and a contrast agent with similar T_1/2_ as the EV turnover (Di Rocco et al., 2016). However, the selection of the imaging modality is not a straightforward decision as there are advantages and limitations associated with each. The major advantage of optical imaging is affordability but the average depth of penetration (mm‐cm range) limits the use of optical systems to tracking EVs in small animals (Stuker et al., 2011; Zinn et al., 2008). Furthermore, absorption and scattering of photons in living tissues hinders the use of optical imaging as a quantitative tool. In contrast, nuclear imaging offers absolute quantification and superb penetration, but is often inaccessible as a research tool due to high cost and lack of instruments in many research facilities. For insightful reviews on the diversity of imaging systems, please refer to Cassidy & Radda (2005) and Yi et al. (2020).

### 4.10 Are we really studying biodistribution of extracellular vesicles?

It is true that individual studies have shown that EVs from different cell sources do possess different biodistribution patterns (Hoshino et al., 2015; Wen et al., 2016; Zhang et al., 2018). We acknowledge that different donor cells may influence the EV biodistribution pattern in individual study settings. However, the holistic analysis shown here revealed that, regardless of the source or size of EVs or the species into which the EVs were delivered, EVs typically accumulate in a restricted number of organs: liver, lungs, kidneys, and spleen. The primary aim of most studies of biodistribution of EVs is to understand which organs/cells the vesicles will affect and to demonstrate where targeted effects of EVs are most likely to occur. We question whether the current studies are actually achieving this aim. It appears to us more likely that the majority of existing studies are showing the uptake of EVs into the major organs of clearance rather than the organs to which EVs are specifically targeted naturally. This is due to experimental constraints that are difficult to modify. Firstly, administration of the EVs was usually via a single bolus which is not reflective of the continuous release of physiological EVs that occurs in vivo. This bolus administration is likely to result in flooding of the animal with EVs and it seems likely that many, if not most of the administered EVs, will not reach their true target before being cleared via the liver and other clearance systems. This is supported by the demonstration that adding specific targeting moieties to EVs increased targeting to specific organs, such as RVG peptide‐expressing EVs being targeted to the brain, but even this manipulation did not appreciably decrease the amount of EVs localized in the liver, spleen, lung, and kidney distribution (Wiklander et al., 2015). Secondly, the sensitivity of the instruments used to detect the EVs is limited. Generally, these instruments are capable of detecting very large accumulations of labelled EVs but it is highly unlikely that such accumulations occur naturally. Thirdly, the dose of EVs that is administered in these studies (often tens to hundreds of μg total protein) is highly likely to far exceed the physiological levels of EVs from any source in vivo. These doses are required in part due to the insensitivity of the equipment used to visualise the EV biodistribution. Furthermore, there is speculation whether fluorescent lipophilic dyes, the most commonly used EV labelling method, could potentially be leaching from EV lipids to cellular membranes without actual EV uptake, giving a false identification of EV internalization (Mulcahy et al., 2014). Provided that the half‐lives of lipophilic dyes are five to > 100 days, this may potentially produce an over‐estimation of EV biodistribution in some organs, particularly in the later timepoints (Chuo et al., 2018). Lastly, different EV isolation methods can yield different biodistribution patterns. For example, IV administration of EVs isolated using ultracentrifugation showed significantly greater detection in the lungs and reduced detection in the liver compared to EVs isolated using ultrafiltration with liquid chromatography (Nordin et al., 2015).

This extreme heterogeneity of experimental parameters emphasizes that the subfield of EV biodistribution is still in its infancy and this was a major challenge when formulating this systematic review and for the field going forward. We believe there would be considerable value in having minimal guidelines for reporting future EV biodistribution studies. We recommend the following list of information to be presented by authors as the minimum requirements for publication of EV biodistribution studies and for guidance in the design of such studies:

- EVs should be characterized and reported in accordance with MISEV guidelines.
- Doses of EVs administered should be reported as at least, total EV protein weight and total particle count. Consideration should be given to the physiological range of EVs in the model.
- EV biodistribution in a wide range of organs should be investigated. To allow comparison between studies, organs to which localization of EVs from diverse sources has been reported e.g., liver, lung, kidneys, and spleen, should be included. Investigators should consider carefully, the full range of controls that are required which might include vehicle only and free‐label controls to rule out false positives from autofluorescence and label‐only biodistribution.
- For those studies where the primary outcome is biodistribution, biodistribution following IV administration should be used as a control for studies investigating non‐IV routes of administration (e.g., IP, SC, IM, OG).
- Methods should indicate whether perfusion of animals to remove circulating EVs were performed prior to imaging.
- Ideally, EV biodistribution should be investigated over a time course. Studies investigating long time‐courses should include analysis at 24 h as a reference point. However, studies employing labelling/contrast agents with short half‐lives (e.g., ^99m^Tc: T_1/2_ = 6 h) should include 1 h as a reference point.
- The raw values detailing EV detection in each organ should be published in some form with the manuscript to allow direct comparison between studies.
- Since there is a clear difference in the biodistribution of EVs between in situ versus ex vivo using fluorescence and bioluminescence‐tracking technologies, consideration should be given to confirming the in situ imaging by ex vivo analysis of organs.

### 4.11 Strengths and limitations

#### 4.11.1 Strengths

This is the first review to systematically assess the literature on the biodistribution of EVs following administration into animals. The PRISMA inspired, unbiased search strategy provides an overview of the relevant literature that is not influenced by the personal experiences or prejudices of the authors (Moher et al., 2009).

The review has somewhat surprisingly identified that holistically, regardless of origin or size of the EVs or the species into which the EVs were administered all EVs localize primarily to four organs; liver, lungs, kidneys, and spleen. This raises the awareness in the potential limitations in the methodology used in this current subfield of EV biodistribution and therefore to considerations that should be given to improving these in future studies.

#### 4.11.2 Limitations

A major limitation of this review is that most of the included studies employed very different experimental parameters, making generalizations difficult. Consequently, detail is lost from the overview that the review provides. In particular, the doses of EVs administered and the timepoints at which the distribution was assessed were very heterogeneous and many studies did not provide thorough characterizations of EVs, including protein markers, electron microscopy, concentration/count measurements. In order to improve the comparability between biodistribution studies, there must be greater effort to standardize these outlined experimental parameters in future studies. Although the MISEV2018 guideline provides excellent recommendations for EV nomenclature, EV separation methods, characterization and functional studies, we hope that future issues will also include recommendations for minimal guidelines for conducting in vivo biodistribution studies (Théry et al., 2018).

Another limitation of this review is the definition of ‘modified’ EVs that we established. As the tracking of EV biodistribution unavoidably requires the artificial labelling of EVs, all studies included in this review involved EVs that were modified in some way, indicating that these EVs were not authentically natural. However, this review made sure to not include any studies that used EVs that were modified in a way to promote selective organo‐tropic behaviour.

While every effort was made to be as inclusive as possible in our search strategy, we cannot guarantee that all relevant studies were captured because they did not contain the keywords used in our search or because they were not listed in the databases we searched.

Lastly, as the search strategy was limited to the English language, there may have been studies that were not captured in this review. Regardless, given the number of studies included, and the heterogeneity of these, it is unlikely non‐English language studies would have altered the results of the review.

## 5 CONCLUSION

In conclusion, understanding the biodistribution of EVs provides a pivotal junction between EV functional studies in vitro and physiological significance. This review highlights that, regardless of the origin or size of EVs, or the species into which the EVs were administered, the biodistribution of EVs was primarily to the liver, lungs, kidneys, and spleen. The distribution to the brain and heart was generally lower and tumour distribution was varied. This review also highlights the extremely heterogeneous experimental parameters used in the current literature which may influence biodistribution of EVs.

## CONFLICTS OF INTEREST

There is no conflict of interest for any of the authors.

## Supporting information

Supporting Information
